# Regulation of Uterine Spiral Artery Remodeling: a Review

**DOI:** 10.1007/s43032-020-00212-8

**Published:** 2020-06-16

**Authors:** Eugene D. Albrecht, Gerald J. Pepe

**Affiliations:** 1 Bressler Research Laboratories, Department of Obstetrics, Gynecology and Reproductive Sciences, University of Maryland School of Medicine, 655 West Baltimore St., Baltimore, MD USA; 2 Department of Physiology, University of Maryland School of Medicine, Baltimore, MD USA; 3 Department of Physiological Sciences, Eastern Virginia Medical School, Norfolk, VA USA

**Keywords:** Uterine artery remodeling, Preeclampsia, Animal models

## Abstract

Extravillous trophoblast remodeling of the uterine spiral arteries is essential for promoting blood flow to the placenta and fetal development, but little is known about the regulation of this process. A defect in spiral artery remodeling underpins adverse conditions of human pregnancy, notably early-onset preeclampsia and fetal growth restriction, which result in maternal and fetal morbidity and mortality. Many in vitro studies have been conducted to determine the ability of growth and other factors to stimulate trophoblast cells to migrate across a synthetic membrane. Clinical studies have investigated whether the maternal levels of various factors are altered during abnormal human pregnancy. Animal models have been established to assess the ability of various factors to recapitulate the pathophysiological symptoms of preeclampsia. This review analyzes the results of the in vitro, clinical, and animal studies and describes a nonhuman primate experimental paradigm of defective uterine artery remodeling to study the regulation of vessel remodeling.

## Introduction

During early human pregnancy, extravillous trophoblast (EVT) migrates to, invades, and replaces the vascular smooth muscle cells (VSMC), endothelial cells, and elastic lamina within, thereby remodeling the uterine spiral arteries [1–4]. Consequently, these arteries change from high-resistance/low-capacity to low-resistance/high-capacity vessels, and thus, uterine artery blood flow increases with advancing gestation to enhance placental perfusion and promote fetal development. Defective uterine artery remodeling (UAR) underpins the etiology of certain pregnancy disorders that comprise the syndrome of placental ischemia [5, 6], notably early-onset preeclampsia, defined as premature delivery prior to week 34 of gestation and associated with a high rate of fetal growth restriction [7–13]. The term preeclampsia is used throughout this review to refer to early onset since in contrast to late-onset preeclampsia, i.e., delivery after 34 weeks, it is underpinned by defective UAR. Preeclampsia is associated with maternal systemic vascular endothelial inflammation-activation-dysfunction, hypertension, renal glomerular endotheliosis, and proteinuria, as well as maternal and neonatal morbidity/mortality [14–20]. It has been proposed that as a consequence of impaired UAR and placental perfusion, the placenta exhibits oxidative stress and the release of anti-angiogenic factors, cytokines, and/or syncytial extracellular vesicles which, along with predisposing maternal factors such as obesity and hypertension, elicit the pathophysiological manifestations of preeclampsia (reviewed in [16, 20]). Excessive trophoblast invasion and UAR are also deleterious because they result in impaired uterine artery vasomotor tone and hemorrhaging after delivery, a pregnancy complication known as placenta accreta [21, 22]. Despite the fundamental importance of UAR to successful pregnancy and fetal development, relatively little is known about the regulation of this process primarily because the majority of studies have focused on the pathophysiological consequences of adverse conditions of pregnancy and not on UAR. The present review describes the results of the in vitro and in vivo studies and a nonhuman primate model to study the regulation of UAR.

## In Vitro Studies

Numerous in vitro studies have been conducted to investigate the ability of primary or immortalized trophoblasts cultured in two or three dimensions to pass across a synthetic permeable membrane coated with matrigel or decellularized extracellular matrix or to form endothelial-like tubes as indices of cell migration and invasion. Collectively, these studies have shown that several factors known to be produced by the placenta and/or decidua, including vascular endothelial growth factor-A (VEGF), placental growth factor (PlGF), insulin-like growth factor (IGF), epidermal growth factor (EGF), heparin-binding EGF (HB-EGF), activin, and human chorionic gonadotrophin (hCG), stimulated HTR-8/SV neo, trophoblast, or choriocarcinoma JEG-3 cell migration or endothelial-like tube formation [23–33]. Moreover, transcription and cell signaling molecules, including the Rac1 member of the Rho family of GTPases, the elastin-derived matrikine VGVAPG, the ephrin-B2 ligand of the Eph receptor, and Notch-2, also increased trophoblast migratory capacity in vitro [34–38]. However, in other in vitro studies, several of these factors did not alter EVT migration [39–41]. In contrast, transforming growth factor (TGFβ)-1, TGFβ-2, and TGFβ-3 and endocrine gland VEGF (EG-VEGF), as well as microRNA-93 and microRNA-135 which decrease CXCL12 gene expression, inhibited migration/invasive capacity of trophoblasts [42–47], while inhibition of TGFβ3 restored invasive capacity of trophoblasts obtained in late gestation from placentas of women with preeclampsia [48]. Additional in vitro studies using placental explants showed that elastin-derived peptides increased and endothelin-1 decreased trophoblast overgrowth [35, 49]. The underlying causes of the divergent effects of these factors on trophoblast migration are unclear, although use of different culture conditions, including oxygen and hypoxia-inducible factor levels and transformed versus primary trophoblasts, may be involved.

The presence of uterine natural killer (uNK) cells and macrophages, which are sources of VEGF-A and VEGF-C, angiopoietins, interleukins, and matrix metalloproteinases (MMPs) [50], was associated with VSMC and endothelial cell disruption in decidual tissue obtained in early human pregnancy [51], while the addition of uNK cell-conditioned medium to cultures of human term chorionic plate arteries caused VSMC and extracellular matrix breakdown [52]. The addition of EVT-conditioned medium to cultures of vascular endothelial cells increased expression of the chemokines CCL14 and CXCL6, which induced chemotaxis of decidual NK cells and macrophages, and the authors proposed that there was crosstalk between EVT, endothelial cells, and decidual immune cells in spiral artery remodeling [53]. NK cells also enhanced migration of and tube formation by primary trophoblast cells from placental villous tips, an effect that was prevented in cultures containing NK cells pretreated with sphingosine FTY720 to suppress NK cell function and VEGF production [54]. Moreover, recent studies suggest that additional processes, including invasion of uterine veins and lymph vessels by endo-venous and endo-lymphatic trophoblast cells, respectively, may also be involved in uterine artery remodeling [55, 56]. Based on these studies, it has been proposed that the immune system plays a role in uterine vessel transformation, although it has been suggested that the role of the immune system is more established in mouse than in human pregnancy (reviewed in [57–59]).

Clearly, the in vitro studies are significant in showing that a multitude of factors have the capacity to alter migratory and invasive capacity of trophoblast cells. However, considering the highly complex interplay of different cell types, molecular events, and spatio-temporal cell interactions that occur in vivo during spiral artery transformation, it is unclear whether trophoblast migration and endothelial tube formation as assessed in vitro validly mirror the process of UAR as it occurs in vivo. Thus, in vivo animal studies are needed to ascertain the applicability and physiological role of the candidate factors shown in vitro either operating alone or in conjunction with each other in regulating UAR.

## Clinical Studies

Human clinical studies have shown that placental expression and/or maternal serum levels of many growth factors, including VEGF, IGF-I, EGF, HB-EGF, TGFβ, soluble endoglin, and other peptides, as well as Notch-2, endothelial colony-forming cells, tyrosine kinase-like orphan receptor, and microRNA-93, are either elevated, decreased, or unaltered in mid to late gestation in women who develop preeclampsia [23, 26, 60–70]. Additional studies have shown that maternal serum levels of PlGF are decreased, and the levels of the sFlt-1 soluble truncated VEGF receptor that binds to and suppresses VEGF bioavailability and endoglin were increased, preceding or coinciding with onset of the complications, e.g., maternal vascular dysfunction, of preeclampsia [71–78]. Consequently, it has been suggested that an imbalance in the levels of anti-angiogenic and angiogenic proteins and other factors may serve as biomarkers that are predictive for early detection of preeclampsia (reviewed in [19, 79, 80]).

Studies have also shown either an increase, no change, or a decrease in maternal serum estradiol levels at mid to late gestation in women exhibiting preeclampsia [81–87]. However, the role of estradiol in early human pregnancy with respect to UAR and onset of the pathophysiological conditions associated with preeclampsia has not been investigated.

Clinical studies have also shown that the number of immune cells, notably uNK cells, macrophages, and dendritic cells is either increased [88–94], decreased [95–97], or not altered [98–100] in decidua/placental bed obtained in late gestation before (e.g., biopsies) or after parturition in patients with preeclampsia. Studies also indicate that women with preeclampsia primarily express the inhibitory and not the stimulatory KIR receptors for uNK cells and that women with a KIR AA genotype, i.e., predictive of expression of the inhibitory KIRs KIR2DL-1, KIR2DL-2, KIR2DL-3, and KIR2DL-5, are at increased risk for developing preeclampsia [101]. Macrophages are also differently activated in preeclampsia [102–106], which may reflect a decrease in M2 macrophages and a concomitant increase in M1 macrophages [92] in the placental bed of preeclamptic women. Such a change would be consistent with increased placental production of pro-inflammatory cytokines [107] and decreased formation of anti-inflammatory cytokines [108, 109] that occur in preeclampsia. Interestingly the levels of mRNAs for immune-associated genes, notably IL-6 and macrophages, as well as markers for expression of M2 macrophages [110] are greater in biopsies of decidua from women in early gestation who subsequently developed pregnancy-induced hypertension compared with those who remained normotensive.

The human studies have been important in correlating the levels of the various factors with the pathophysiological features of preeclampsia. However, it is difficult to test cause and effect and the alterations in the various factors at mid-late gestation in preeclampsia patients may result from and not underpin the pathophysiological conditions elicited by preeclampsia. Importantly, since UAR was not simultaneously examined in these clinical studies, the regulatory role of these factors on UAR has not been established in normal or adverse human pregnancy.

## Animal Studies

**Fig. 1:**
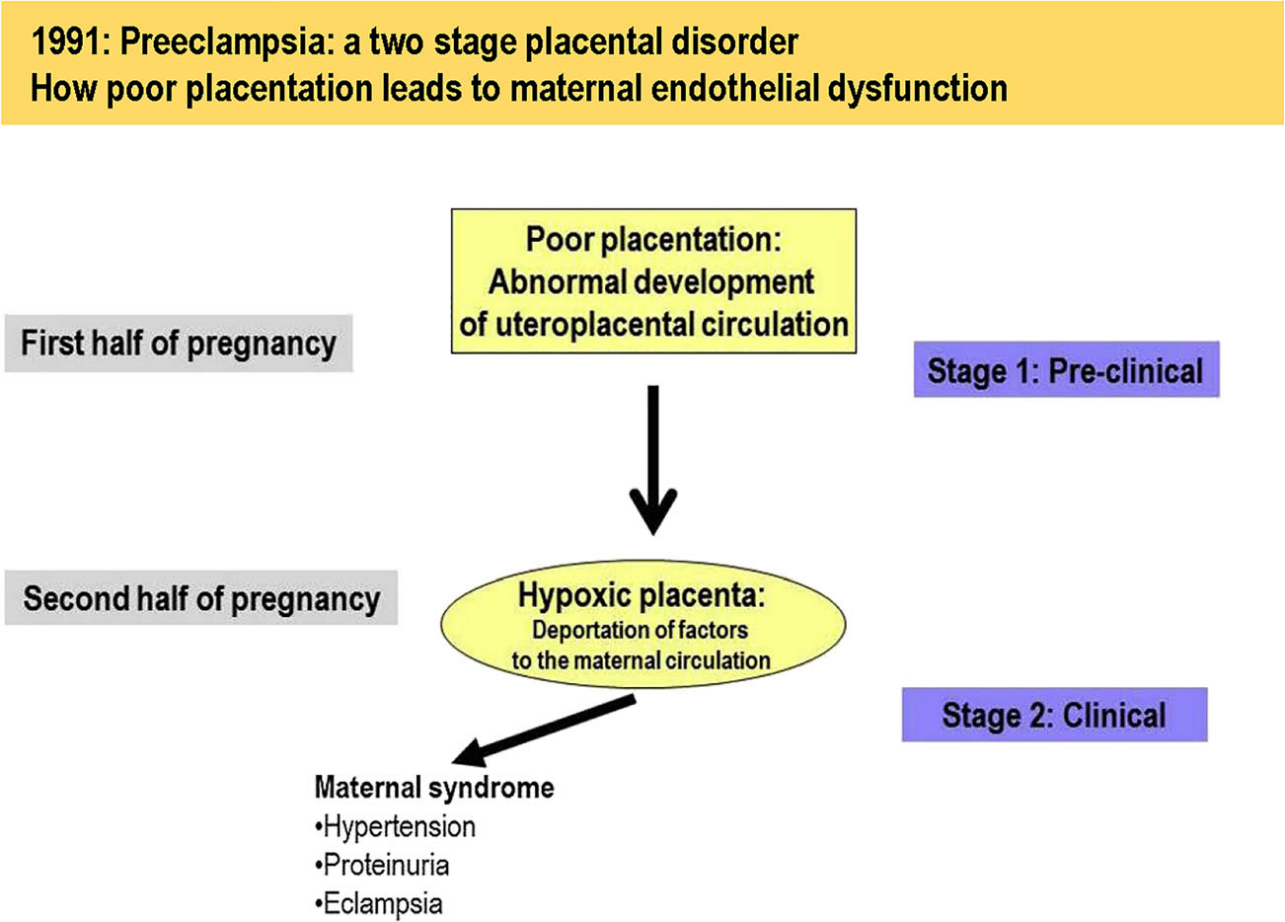
The two-stage placental model of preeclampsia in which it has been proposed [111] that impaired remodeling of uterine spiral arteries (“poor placentation”) is the pathway to stage 1 preeclampsia (placental dysfunction) and the preclinical stage before development of the maternal clinical syndrome (stage 2). Reprinted from Staff [20]

**Fig. 2:**
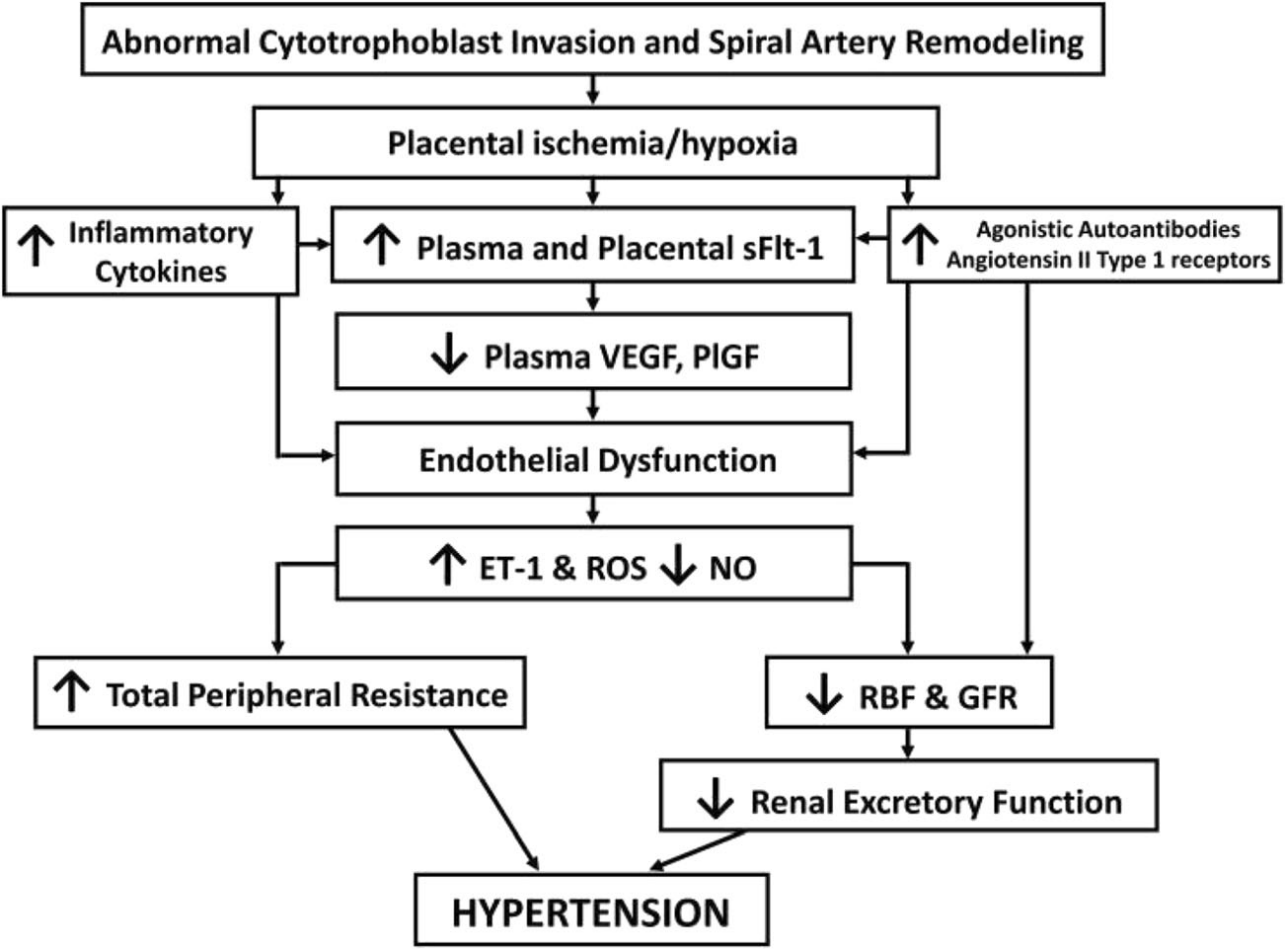
Hypothetical scheme depicting how abnormal trophoblast invasion and spiral artery remodeling result in placental ischemia, endothelial dysfunction, and hypertension in preeclampsia. Reprinted from Palei et al. [17]

As presented in recent reviews [16, 19, 20, 111], early-onset preeclampsia is considered a two-stage disorder, stage 1 reflecting reduced placental perfusion and dysfunction due to impaired UAR and stage 2 the maternal syndrome induced by inadequate placental perfusion and deportation into the maternal blood of placental factors and syncytial particles produced in response to placental hypoxia and oxidative stress (Fig. 1). Although the maternal disorder including organ system involvement can vary greatly in complexity and severity [16, 111], maternal systemic vascular dysfunction and hypertension are hallmark features of preeclampsia. These manifestations appear to result from vascular endothelial inflammation, oxidative stress and dysfunction, notably impaired ability to produce the vasodilators nitric oxide (NO) and prostacyclin I_2_, increased production of vasoconstrictors such as endothelin, and hyper-sensitivity of VSMC to vasoconstrictors within the vascular bed (Fig. 2) [12, 14, 16–18, 20, 112–121].

It is well established that VEGF plays a pivotal role in promoting vascular endothelial cell integrity, stability, and function, including NO production [122–124]. Therefore, it has been hypothesized [19, 75, 117] that in preeclampsia the placental ischemia induced by defective UAR causes an increase in placental expression and maternal serum levels of sFlt-1, which decreases VEGF bioavailability and elicits maternal vascular dysfunction (Fig. 2). Accordingly, animal models have been developed to examine this hypothesis and ascertain the possibility of achieving a therapeutic approach to overcome or prevent the vascular dysfunction elicited by decreased bioavailability of VEGF. Thus, key manifestations of preeclampsia, i.e., maternal hypertension, fetal growth restriction, and/or maternal vascular endothelial dysfunction, were induced in mice or rats in which levels of sFlt1 and/or endoglin were elevated by systemic adenoviral delivery of these proteins [74, 125–131]. Systemic administration to mice of an antibody which neutralized both Flt-1 and sFlt-1 decreased uterine artery length as an index of arterial transformation in this species [132]. The clinical manifestations of preeclampsia elicited in several of these animal models were prevented by adenoviral delivery of VEGF_121_ [133–138]. Symptoms of preeclampsia were also overcome in lentiviral sFlt1-treated mice by concomitant administration of the drug pravastatin [139] and in BPH/5 mice by injection of the drug celecoxib at the time of embryo implantation, which apparently acted by restoring the levels of Cox 2, VEGF, and related angiogenic factors [140]. Mice defective for PlGF, a member of the VEGF family, also exhibit preeclampsia-like symptoms, notably maternal endothelial dysfunction, as well as cognitive function of the offspring [141, 142]. Moreover, an experimental increase in sFlt-1 levels or decrease in VEGF and PlGF levels induced in rats and sheep by aortic or uterine artery ligation to elicit placental ischemia caused maternal hypertension, proteinurea, and vascular dysfunction, effects reversed by VEGF or PlGF administration [135, 138, 143–145]. Uterine spiral arteriole remodeling and MMP-2 and MMP-9 were decreased in the rat reduced uterine perfusion pressure model [146].

Inhibition of NO synthase [147], as well as administration of tumor necrosis factor-α [143] or interleukin [148], also induced preeclampsia-like symptoms in mice and rats. Interestingly, uterine artery diameter and length were reduced in endothelial NO synthase-null mice [149], whereas nanoparticle-mediated delivery of the NO donor SE175 to mice at mid-late gestation increased spiral artery diameter [150]. Roles for the Notch signaling pathway and the storkhead box 1 (STOX 1) transcription factor have also been suggested since Notch 2-null mice exhibited a decrease in spiral artery diameter and placental perfusion [151], while overexpression of STOX 1 in mice led to a preeclampsia phenotype of hypertension [152].

The role of immune cells in the process of vessel transformation has been proposed. Thus, studies in uNK cell-immunodeficient mice indicate that uNK cells, via the formation of interferon gamma, promote modification (i.e., luminal area) of spiral arteries [153–156]. Moreover, T lymphocyte regulatory cell (Tregs)-deficient mice show impaired uterine artery remodeling and flow [157, 158], suggesting that Tregs impair inflammatory responses that cause a defect in uterine vessel transformation [159].

Evidence for involvement of the renin-angiotensin (AT)-aldosterone system in preeclampsia has also emerged from rodent models. Thus, administration of antibodies to AT_1_ beginning at midgestation to mice or rats elicited hypertension, proteinuria, glomerular endotheliosis, and placental abnormalities [160, 161]. Moreover, AT_1_-deficient mice exhibited impaired placentation [162], and angiotensinogen transgenic mice exhibited deeper endovascular trophoblast invasion and spiral artery remodeling [163]. Upregulation of VSMC AT_1_ expression elicited hypertension, proteinuria, increased sFlt-1 expression, and decreased placental labyrinth growth in mice, effects prevented by administration of β-arrestin, a G protein that causes AT_1_ receptor desensitization [164].

The rodent models have been valuable in recapitulating the clinical symptoms of pregnancy disorders such as preeclampsia. However, in most instances, UAR was not examined, experimental interventions used to induce preeclampsia-like symptoms were often applied after the time of placentation, and many of the clinical features of preeclampsia were also induced in nonpregnant rodents, and thus, these models were not specific for pregnancy. Moreover, there are significant differences in placental morphology and development, the process and impact of spiral artery remodeling, uterine and placental vascular anatomy, and the maternal-placenta-fetal endocrine inter-relationships between rodents and humans [58, 59, 165–171]. For example, in the mouse and rat, trophoblast invasion is temporally restricted to late gestation [58, 172] and the role of UAR on maternal vascular function may be equivocal. Thus, although NK-defective mice exhibit impaired UAR, maternal resting blood pressure remains normal throughout gestation and maternal proteinuria does not develop [155], while trophoblast arterial invasion is more extensive and uterine artery resistance lower in the rat BHP/5 model of preeclampsia [163]. Collectively, these differences between rodents and humans make translation of findings on UAR in the rodent to the human uncertain.

Although rodents have been extensively used to recapitulate the pathophysiological features of preeclampsia, relatively few studies have employed nonhuman primates in this area of perinatal biology. Placental morphology, the process of uterine spiral artery transformation, uterine and placental vascular anatomy, and maternal-placental-fetal endocrine inter-relationships are similar in human and baboon pregnancy [58, 165, 173]. Although remodeling of the spiral arteries in the baboon does not extend into the inner myometrium, as in human pregnancy, the qualitative nature of placentation and UAR are alike [58, 174]. In addition to these important considerations, humans and baboons exhibit similar anatomy, physiology, and ontogeny of the fetal-placental unit [165] and share > 96% DNA/genetic homology [175, 176], and thus, the baboon provides an excellent nonhuman primate model for the study of human placental and fetal development.

As in the rodent studies, uterine artery ligation has been employed as an experimental paradigm in pregnant baboons. Uteroplacental ischemia elicited by uterine artery ligation in the second half of baboon pregnancy resulted in hypertension, proteinuria, and renal endotheliosis, effects reversed by administration of sFlt-1 siRNA or PlGF [177–180]. Thus, the latter primate studies focused on recapitulating the symptoms of adverse human pregnancy but not on UAR.

**Fig. 3:**
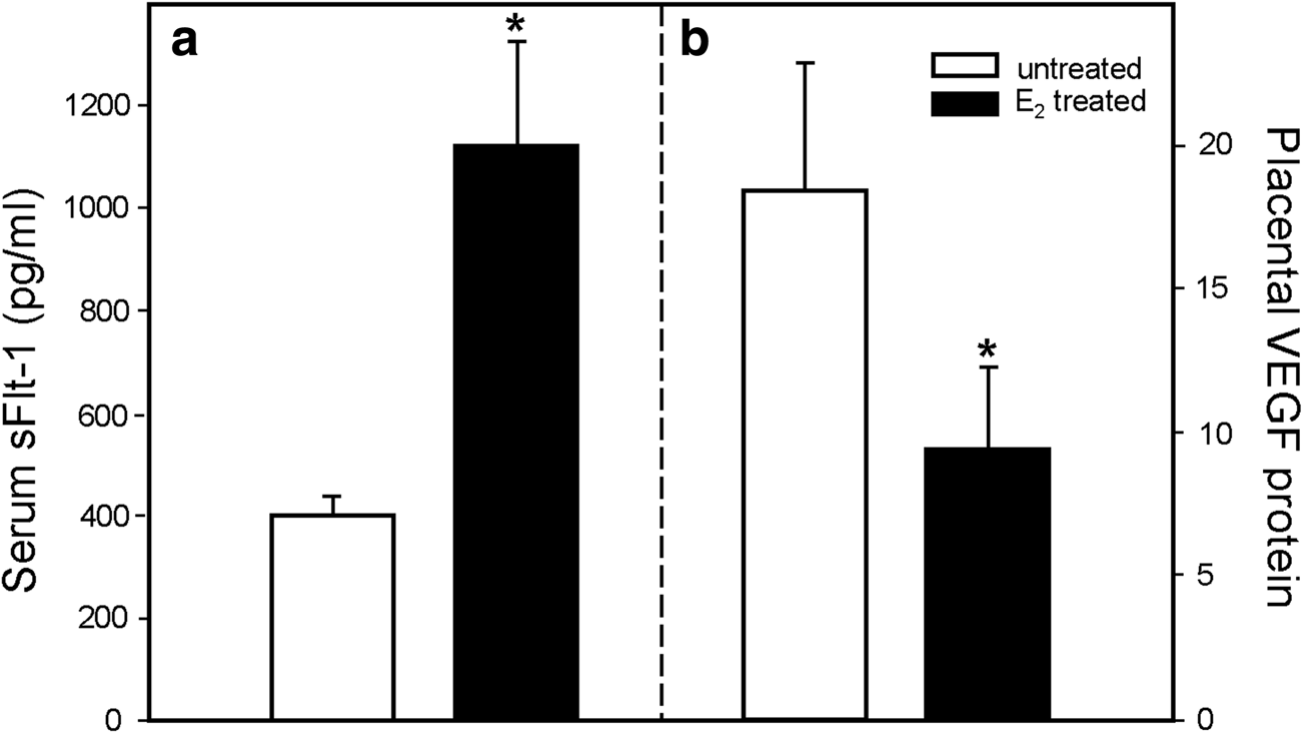
(**a**) sFlt-1 levels in uterine vein and (**b**) VEGF protein quantified by proximity ligation assay (signals/nuclear area × 10^4^) in the anchoring villi on day 60 in untreated and estradiol (E_2_)-treated baboons. *P < 0.05

**Fig. 4:**
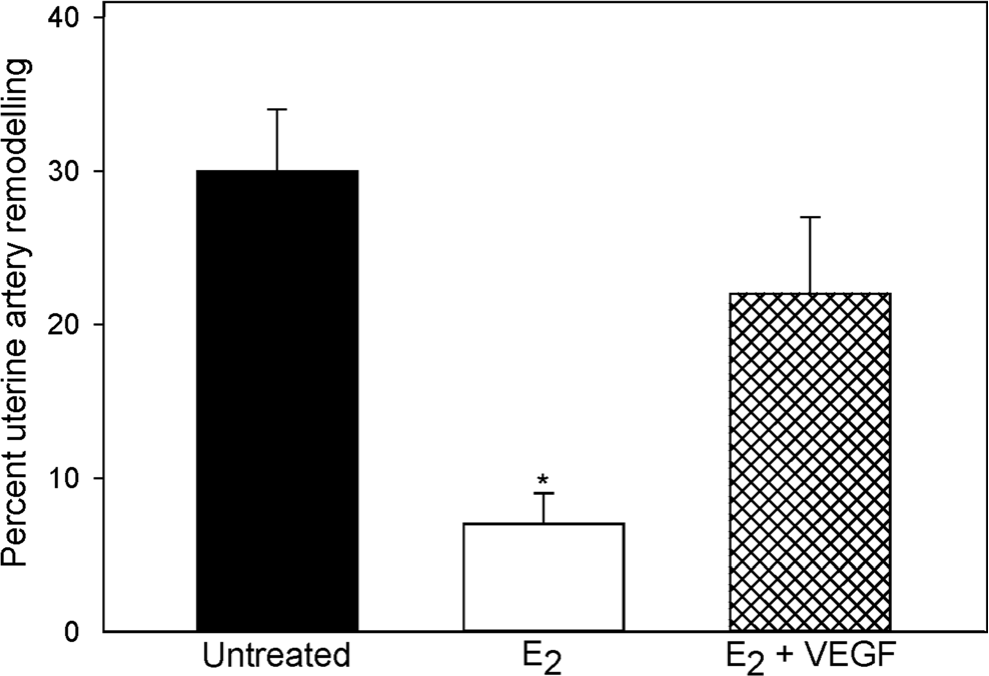
Percent remodeling of uterine spiral arteries (i.e., number of vessels exhibiting trophoblast invasion divided by total number of vessels counted) on day 60 of gestation in baboons untreated, treated with estradiol (E_2_), or treated with E_2_ plus VEGF DNA. *Different (*P* < 0.01) from values in other two groups

In contrast to the latter approach, the authors have published a series of studies in which they have established an experimental paradigm of prematurely elevating estradiol levels in the first trimester of baboon pregnancy to study the regulation of UAR [181–183]. Slightly elevating maternal estradiol levels resulted in a 3-fold increase in placental expression and maternal serum levels of sFlt-1 and decrease in extravillous trophoblast expression of VEGF in early pregnancy (Fig. 3). The increase in sFlt-1/decrease in VEGF was associated with a 75% reduction in the level of UAR, quantified as the percent of uterine spiral arteries invaded and remodeled by extravillous trophoblasts, at the end of the first trimester (Fig. 4). Concomitant administration of estradiol and delivery of the VEGF gene selectively to the maternal aspect of the placenta, but not the fetus, by contrast-enhanced ultrasonography/microbubble technology restored VEGF protein levels and prevented the decrease in UAR (Fig. 4, [184]).

The decline in extravillous trophoblast VEGF expression in estradiol-treated baboons was associated with a decrease in expression of the α_1_β_1_ and α_5_β_1_ integrins [182] that promote trophoblast migration and remodeling and are increased by VEGF in vitro [185–187]. This suggests that these integrins may mediate the stimulatory effect of VEGF on UAR during early baboon pregnancy. Coinciding with the decrease in UAR, uterine artery blood flow was reduced by 30% and maternal blood pressure increased by 25% near term, suggesting an impairment of maternal systemic vascular function [183]. Although it has been suggested that the alteration in expression of pro- and anti-angiogenic growth factors is simply the result and not the cause of placental dysfunction in preeclampsia [20], the prevention of the decrease in UAR by VEGF delivery in early baboon pregnancy is consistent with VEGF having a pivotal role in promoting UAR.

## Summary

UAR is vital to successful pregnancy; however, the regulation of this fundamentally important process has not been established. The in vitro studies are important in having identified a multitude of factors that have the ability to alter migratory and invasive capacity of trophoblast cells. However, it is unclear whether trophoblast migration and endothelial tube formation as assessed in vitro validly mirror the process of UAR as it occurs in vivo. The clinical studies have been significant in showing that maternal serum levels of certain factors are altered, particularly sFlt-1 which is increased and PlGF which is decreased, preceding or coinciding with onset of the complications, e.g., maternal vascular dysfunction, emanating from preeclampsia. However, it is difficult to test cause and effect in human pregnancy studies, and thus, the alteration in circulating levels of the various factors may be a consequence of and not underpin the pathophysiological conditions elicited by adverse pregnancy. The rodent and a few primate studies have been valuable in recapitulating, and showing the ability of certain growth factors to mitigate, the clinical manifestations of pregnancy disorders such as preeclampsia, but have not focused on UAR. This review has described the results of in vitro, clinical, and rodent studies and also a novel experimental model of defective UAR in a nonhuman primate that allows study of the regulation of spiral artery transformation and the potential to develop therapeutic modalities to manage or prevent the maternal pathophysiological consequences of adverse pregnancy arising from defective UAR.
